# A novel dual epigenetic approach targeting BET proteins and HDACs in Group 3 (MYC-driven) Medulloblastoma

**DOI:** 10.1186/s13046-022-02530-y

**Published:** 2022-11-11

**Authors:** Matthew J. Kling, Varun Kesherwani, Nitish K. Mishra, Gracey Alexander, Erin M. McIntyre, Sutapa Ray, Kishore B. Challagundla, Shantaram S. Joshi, Don W. Coulter, Nagendra K. Chaturvedi

**Affiliations:** 1 Department of Oral Biology, Creighton University, School of Dentistry, Omaha, NE 68102 USA; 2 Child Health Research Institute, University of Nebraska Medical Center, Omaha, NE 68198 USA; 3 Department of Genetics, Cell Biology and Anatomy, University of Nebraska Medical Center, Omaha, NE 68198 USA; 4 Department of Pediatrics, Hematology/Oncology Division, University of Nebraska Medical Center, Omaha, NE 68198 USA; 5 Department of Biochemistry and Molecular Biology, University of Nebraska Medical Center, Omaha, NE 68198 USA; 6 Department of Pediatrics, Hematology and Oncology Division, University of Nebraska Medical Center, Nebraska Medical Center, Omaha, NE 68198 USA

**Keywords:** Medulloblastoma, MYC, BET proteins, HDACs, BET-HDAC inhibitors, Gene transcription

## Abstract

**Background:**

Medulloblastoma (MB) patients with MYC oncogene amplification or overexpression exhibit extremely poor clinical outcomes and respond poorly to current therapies. Epigenetic deregulation is very common in MYC-driven MB. The bromodomain extra-terminal (BET) proteins and histone deacetylases (HDACs) are epigenetic regulators of MYC transcription and its associated tumorigenic programs. This study aimed to investigate the therapeutic potential of inhibiting the BET proteins and HDACs together in MB.

**Methods:**

Using clinically relevant BET inhibitors (JQ1 or OTX015) and a pan-HDAC inhibitor (panobinostat), we evaluated the effects of combined inhibition on cell growth/survival in MYC-amplified MB cell lines and xenografts and examined underlying molecular mechanism(s).

**Results:**

Co-treatment of JQ1 or OTX015 with panobinostat synergistically suppressed growth/survival of MYC-amplified MB cells by inducing G2 cell cycle arrest and apoptosis. Mechanistic investigation using RNA-seq revealed that co-treatment of JQ1 with panobinostat synergistically modulated global gene expression including MYC/HDAC targets. *SYK* and *MSI1* oncogenes were among the top 50 genes synergistically downregulated by JQ1 and panobinostat. RT-PCR and western blot analyses confirmed that JQ1 and panobinostat synergistically inhibited the mRNA and protein expression of MSI1/SYK along with MYC expression. Reduced SYK/MSI expression after BET (specifically, BRD4) gene-knockdown further confirmed the epigenetic regulation of *SYK* and *MSI1* genes. In addition, the combination of OTX015 and panobinostat significantly inhibited tumor growth in MYC-amplified MB xenografted mice by downregulating expression of MYC, compared to single-agent therapy.

**Conclusions:**

Together, our findings demonstrated that dual-inhibition of BET and HDAC proteins of the epigenetic pathway can be a novel therapeutic approach against MYC-driven MB.

**Supplementary Information:**

The online version contains supplementary material available at 10.1186/s13046-022-02530-y.

## Background

Medulloblastoma (MB) is the most common type of childhood brain cancer worldwide [1]. Treatment for MB includes surgery, radiation, and chemotherapy. Unfortunately, current treatments are associated with long-term morbidity, including lifelong cognitive deficiencies, endocrine dysfunction, neurological deficits, and secondary tumors [1, 2]. MB has biological/genetic heterogeneity with classically four major molecularly distinct subgroups that include wingless (WNT), Sonic-hedgehog (SHH), Group 3, and Group 4 [3, 4]. The 2021 WHO Classification of Tumors of the Central Nervous System classified MB into four molecularly defined MBs (WNT-activated, SHH-activated and TP53-wildtype, SHH-activated and TP53-mutant, non-WNT/non-SHH) and one histologically defined group [5]. In this classification, non-WNT/non-SHH comprised Group 3 and Group 4 tumors. Of these, Group 3 MB often exhibits MYC amplification (17–20% of cases) or overexpression and has the worst prognosis of the MB subgroups, with < 60% 5-year overall survival. MYC-driven MB has high metastatic potential and is often resistant to even multimodal treatments [6–8]. There is a pressing need to develop novel targeted therapies that attack Group 3 MB while incurring limited toxicities.

Epigenetic mechanisms are increasingly considered major factors contributing to MB pathogenesis [9, 10]. Particularly, dysregulation of epigenetic/chromatin modifiers, including histone acetylation/methylation marks, is very common in in Group 3 and Group 4 MBs, compared with other subgroups [11, 12]. Histone acetylation marks play important roles in regulating gene transcription, including transcription of *MYC* oncogene. These marks are written by histone acetyltransferases (HATs), erased by histone deacetylases (HDACs) and read by bromodomain extra-terminal (BET) proteins [13–15]. Importantly, HDACs and BET proteins have been shown to regulate MYC transcription and its associated tumorigenic programs [16, 17], making them rational targets for MYC-driven MB therapy.

HDACs have been implicated in promoting tumorigenesis mainly by silencing tumor suppressor genes and apoptosis inducers [18]. As HDACs are frequently overexpressed in cancers including MB, they have been recognized as promising therapeutic targets, and several pharmacologically distinct HDAC inhibitors have been developed [19, 20]. Various HDAC inhibitors, such as panobinostat and vorinostat, have shown potent efficacy against MB in preclinical studies [21, 22]. More than a dozen HDAC inhibitors are currently being evaluated in the clinic and/or clinical trials [20]. While the results of preclinical studies are encouraging, early clinical trials of HDAC inhibitors have only shown a limited efficacy in patients with advanced tumors. Therefore, it is important to explore epigenetic-based drug combination strategies to improve efficacy against tumors in general, and to identify promising approaches against MB in particular.

As chromatin readers, the BET proteins (such as BRD3 and BRD4) bind to acetylated lysine residues on histone proteins and play important roles in the transcription of oncogenes such as *MYC* and *MYCN* [23, 24]. BET inhibitors, such as JQ1 and OTX-015, competitively bind to acetyl–lysine recognition pockets, displace BET bromodomain proteins from chromatin, and inhibit the expression of oncogenes, leading to cancer cell growth inhibition and apoptosis. BET inhibitors have shown promising *in vitro* and *in vivo* anticancer effects against MB, NUT-midline carcinoma, multiple myeloma, lymphoma, leukemia, and neuroblastoma [16, 23–25]. However, treatment with BET protein inhibitors alone does not cause cancer remission in MB-bearing mice [16].

Synergistic effects of HDAC and BET inhibitors have been observed in different cancer types, including neuroblastoma and glioblastoma [26–29]. However, concurrent targeting of BET proteins and HDACs in MB remains unexplored. In this study, we tested the effects of BET inhibitor and HDAC inhibitor co-treatment on global gene expression, including *SYK* and *MSI1* oncogenes; MYC protein expression; and anticancer efficacy against MYC-driven MB *in vitro *and *in vivo*.

## Methods

### Cell lines and small molecule inhibitors

MB cell lines D-283 (MYC-amplified) and D-341(MYC-amplified) were purchased from American Type Culture Collection. HD-MB03 (MYC-amplified) MB cell line was purchased from Deutsche Sammlung von Mikroorganismen und Zellkulturen (Germany). ONS-76 (non-MYC-amplified, SHH) MB cell line was purchased from Sekisui-XenoTech (USA). Cell lines were authenticated by their respective resources using short tandem repeat profiling. All cell lines were also tested for negative mycoplasma contamination using MycoSensor-PCR assay kit (Agilent-Technologies). All cell lines were cultured and maintained using RPMI-1640 media supplemented with 10% heat-inactivated FBS, penicillin (100 U/ml) and streptomycin (100 µg/ml) (Life Technologies) in a humidified incubator at 5% CO2 and 95% air atmosphere at 37 °C. Experiments were performed using less than 10 passages for each cell line. The BET-protein inhibitors JQ1 and OTX-015 and pan-HDAC inhibitor panobinostat were purchased from MedChemExpress LLC.

### Cell growth, apoptosis, and cell cycle analyses

Cell growth, apoptosis, and cell cycle analyses in MB cells treated with inhibitors were performed using the MTT assay, Annexin-V assay and propidium-iodide staining, respectively, as described previously [30–32].

### Western blotting

Western blot analysis was performed using a previously described protocol in our lab [30]. Primary antibodies used in this analysis included c-MYC, BRD4, SYK, MSI1, Cyclophillin B and Vinculin (Cell Signaling Technology).

### Quantitative RT-PCR (qRT-PCR)

RNA was prepared using RNeasy (Qiagen) kit and 2 µg of total RNA was used for cDNA preparation using Superscript Verso enzyme kit (Promega). cDNA product was amplified in 10 µl reaction using SYBR-Green Super-mix and standard gene-specific primers (Applied Biosystems). In this analysis, pre-designed primers for *SYK*, *MSI1* and *MYC* genes from Integrated DNA Technologies (IDT), were used. All reactions were processed in a QuantStudio-3 PCR System and results analyzed by QuantStudio software (Applied Biosystems).

### siRNA knock-down and transfection

All SMARTpool siRNAs (a pool of 3–4 target-specific siRNAs of each gene) used were purchased from Dharmacon Inc. Non-targeting control (NTC) (D-001810–10-05), BRD4 (L-004937–00-0005), SYK (L-003176–00-0005) and MSI1 (L-011338–00-0005) siRNAs (each at 50 nM) were transiently transfected into MB cells using Lipofectamine^2000^ (Invitrogen) according to the manufacturer’s instructions. Following 72 h of transfection, cells were subjected to downstream analyses using western blotting, q-PCR and MTT assays.

### RNA sequencing and gene expression analyses

RNA from inhibitor-treated HD-MB03 cells, was purified using the Qiagen RNeasy Kit. After confirming sequence grade quality of RNA using an Agilent 2100 Bioanalyzer, an RNA library was prepared using True-Seq RNA Sample Prep V2 Kit and subjected to RNA sequencing using the Illumina NextSeq550 system in the UNMC Genomics Core Facility. Each sample was processed in triplicate. In this analysis, approximately 22,000 protein coding genes were sequenced. The original fastq reads were processed by a newly developed standard pipeline utilizing STAR as the aligner and RSEM as the tool for annotation and quantification at both gene and isoform levels. Using these reads, the normalized FPKM and TPM values for all the available genes were calculated and then differential gene expression and gene-set-enrichment (GSE) analysis between treatment groups were performed. The defined gene sets for MYC/HDAC and other cancer hallmark targets used in this study were from The Molecular Signatures Database (MSigDB).

### Animal studies

All animal experiments were performed according to a UNMC Institutional Animal Care and Use Committee (IACUC) approved protocol. In this study, six- to eight-week-old NSG female mice from Jackson Laboratories were used. We used only female mice to limit biological variables. These mice were subcutaneously injected in the right-flank with 2.5 × 10^5^ HD-MB03 MB cells suspended in 100 µl of a 3:1 PBS/Matrigel mixture. Ten days post-tumor injection, when tumor was palpable, the tumor bearing mice were divided into four treatment groups (*n* = 5 each group) and treated three times a week for three weeks. The vehicle control treatment was (5% DMSO + 45% PEG 300 + 2% Tween 80. Experimental treatments were OTX-015 (50 mg/kg, p.o.), panobinostat (10 mg/kg, i.p.), or the combination of OTX-015 with panobinostat. The vehicle control was administered using both intraperitoneal (i.p.) and oral (p.o.) routes. The OTX015 and panobinostat were dissolved in a small volume (50 µl) of 100% DMSO solvent and stored at -20 °C. These inhibitors were further diluted with PEG 300 and Tween 80 accordingly to make DMSO concentration to 5% in a final treatment solution. Doses for these inhibitors were at ranges of achievable exposures in mice or humans [33–36]. Tumor volume was assessed twice a week using the digital caliper. When tumor volume approached 2 cm^3^, the mice were euthanized using CO_2_ and tumor tissues were collected and processed for immunohistological analyses for the expression of key proteins.

### Statistical analysis

All experiments were repeated at least three times and mean and standard error values were calculated. Statistically significant differences were calculated using Student t-tests or analysis of variance (ANOVA) and a significance threshold of *p* < 0.05 or *p* < 0.01, as noted. To determine synergy, we employed the Chou and Talalay method for combination index (CI) analysis using CalcuSyn software (Biosoft, UK) [37]. CI < 0.9 indicates synergism, 0.9–1.1 additivity and > 1.1 antagonism.

## Results

### Synergistic effects of the inhibitors of BET proteins and HDACs on MB cell growth

Because BET inhibitors inhibit the expression of tumor promoting genes and HDAC inhibitors induce the expression of tumor silencing genes, we explored the synergistic anticancer effects of BET inhibitors (JQ1, OTX015) and an FDA approved pan-HDAC inhibitor panobinostat on the cell growth of MYC-amplified and non-MYC-amplified MB cell lines using a standard MTT assay. We have previously shown in one of our MB studies that MYC-amplified cell lines are more sensitive to BET inhibitors [31], compared to non-MYC MB cell lines, suggesting that BET inhibitors can synergize with other targeted agents (such as HDAC inhibitors) more efficiently in MYC-amplified MB cells. In this i*n vitro* study, low-µM or nM concentrations of these inhibitors were employed as previously standardized and reported by us and others [26, 27, 31].

**Fig. 1:**
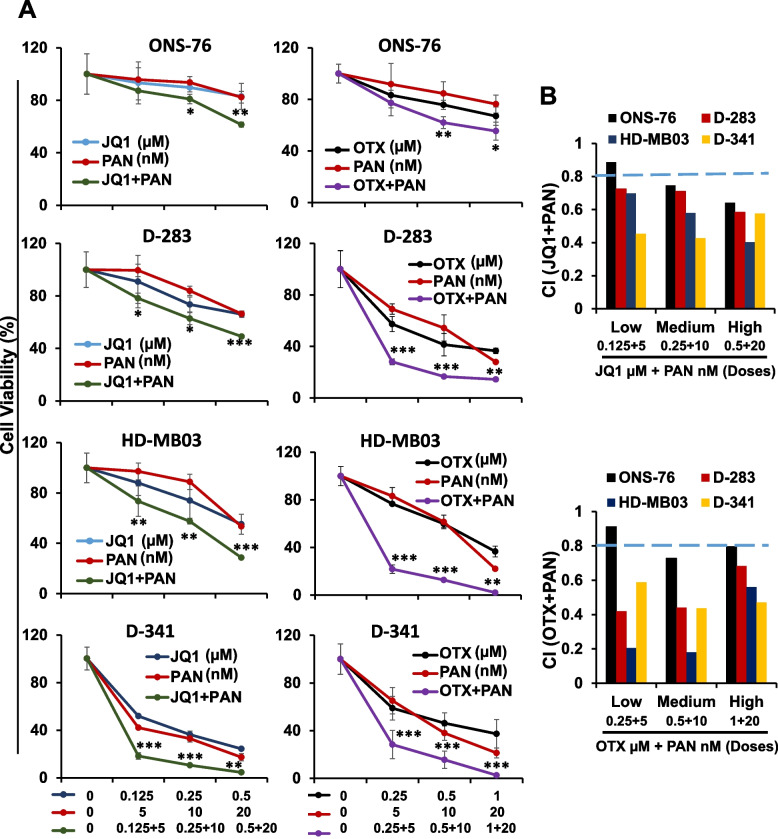
Synergistic effects of BET and HDAC inhibitors on MB cell growth. **A** MTT assay showing dose-dependent growth effects of BET inhibitors JQ1 or OTX015 (OTX) and HDAC inhibitor panobinostat (PAN) alone or combined, with the doses as indicated, in MB cell lines at 72 h. Percentage of viable cells is relative to DMSO-(solvent control)-treated cells. Results are the representative of three independent replicates. Plotted values and error bars represent mean ± SEM. *p < 0.05; **p < 0.01; ***p < 0.005 (Student-*t*-test). The asterisks are showing significance between single agents and combinations (JQ1 or PAN vs JQ1-PAN combination; OTX or PAN vs OTX-PAN combination). **B** Combination index (CI) analysis for the synergism of JQ1/PAN or OTX/PAN in MB cell lines

We tested the single agents and combinatorial/synergistic potential of BET inhibitors (JQ1 or OTX015) and HDAC inhibitor panobinostat, in MB cell lines, including three MYC-amplified (D-283, HD-MB03, D-341) and one non-MYC-amplified (ONS-76). These were treated with increasing concentrations of JQ1, OTX015 and panobinostat, alone or in combinations with either JQ1-panobinostat or OTX015-panobinostat, for 72 h. The MTT results (Fig. 1A) showed a dose-dependent cell growth inhibition of all MB cell lines by BET inhibitors (JQ1 or OTX015) at low-µM and panobinostat at nM concentrations. At lower doses, each inhibitor displayed superior efficacy in MYC-amplified lines, compared to non-MYC-amplified MB cells. Interestingly, co-treatment of JQ1 or OTX015 with panobinostat significantly suppressed growth of MYC-amplified MB cell lines in a dose-dependent manner, compared with single agent treatment (Fig. 1A). Although there was a significant effect of this combination in non-MYC MB (ONS-76) cells treated with higher doses, growth inhibition was more pronounced in MYC-amplified MB cells, consistently suggesting MYC-dependent efficacies of these inhibitors. Combination index (CI) analysis by the Chou-Talalay method [37] confirmed that combinations of BET-HDAC inhibitors had strong and greater synergistic inhibitory effects on MYC-amplified MB cell growth, with CI values ranging from 0.2 to 0.7 in MYC-amplified cell lines and 0.7 to 0.9 in non-MYC MB cells (Fig. 1B). Together, results suggested synergistic anti-MB potential of BET-HDAC inhibition.

### Combination of BET inhibitors with panobinostat induces cell cycle arrest and apoptosis

**Fig. 2:**
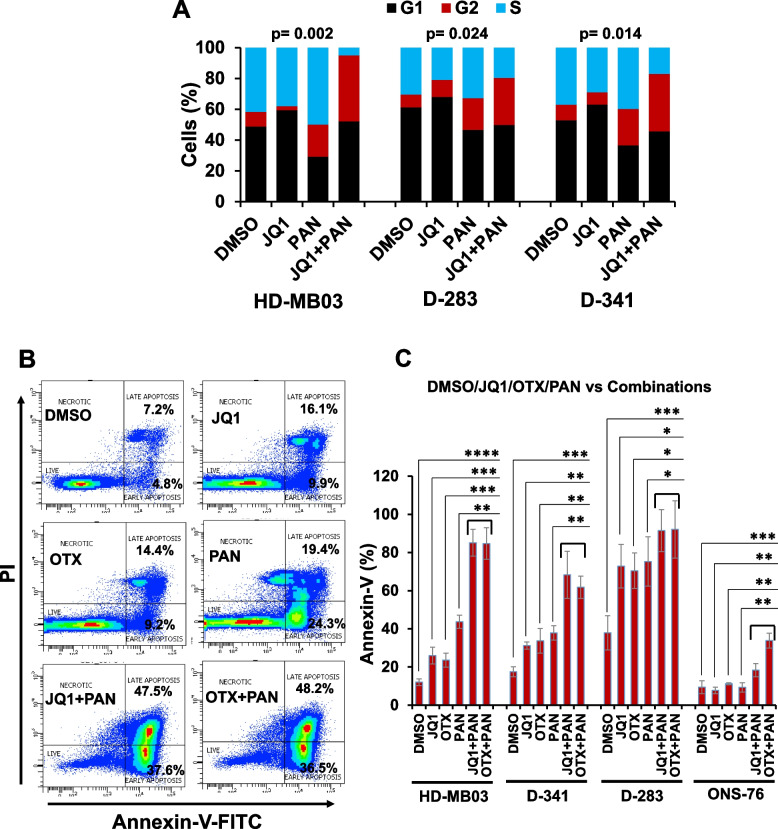
Combination of BET and HDAC inhibitors leads to cell cycle arrest and induces apoptosis. **A** Cell cycle analysis of three MYC-amplified MB cell lines (HD-MB03, D-341, D-283) using propidium-iodide (PI) staining following treatment with JQ1 (0.5 µM) and PAN (10 nM) alone or combined for 24 h. Cumulative Chi Square (χ2)-derived p-value for the cell cycle distribution in each cell line with treatments. This p-value denotes significance between all treatments. **B** Representative flow cytometry-derived scatter diagrams of Annexin-V-FITC staining show apoptosis induction in MYC-amplified HD-MB03 cells treated for 72 h with 0.5 µM JQ1 or OTX, 10 nM PAN alone, or combinations of PAN with JQ1 or OTX. **C** Bar graphs show quantification of Annexin-V/PI double positive apoptotic cells in three MYC-amplified (HD-MB03, D-341, D-283) and one non-MYC-amplified (ONS-76) MB cell lines treated as above. Results are the representative of three independent experiments. Plotted values and error bars represent mean ± SEM. *p < 0.05; **p < 0.01; ***p < 0.005; ****p < 0.001 (Student-*t*-test). The p-values denote significance of cumulative comparison between DMSO-control or single agents and combinations

To determine if combination of BET inhibitors with panobinostat induces cell cycle arrest and apoptosis, MB cell lines were treated with a sub-optimum (IC_50_) concentration of each inhibitor, alone or combined and subjected to cell cycle analysis using propidium-iodide staining and apoptosis analysis using Annexin-V staining. The cell cycle profile (Fig. 2A) in MYC-amplified (D-283, D-341, HD-MB03) cell lines showed that while JQ1 arrested the cells in G1 phase and panobinostat in G2 phase of the cell cycle, co-treatment of JQ1 and panobinostat drastically increased the population of cells in G2 phase compared to individual treatments. These results suggest that as single agents, JQ1 and panobinostat target cell cycle differently, but in combination, JQ1 has an additive effect on panobinostat-induced G2 cell cycle arrest. Results of apoptosis analyses (Fig. 2B and 2C) using an Annexin-V assay demonstrated significantly increased induction of apoptosis after combined treatment with BET inhibitor (JQ1 or OTX015) and panobinostat in all MB cell lines, compared to single agents. There was also significant induction of apoptosis by JQ1, OTX015 or panobinostat alone in MYC-amplified (D-283, D-341, HD-MB03) cell lines. While the combination of BET-HDAC inhibitors significantly induced apoptosis in non-MYC (ONS-76) cells, we did not observe any significant effects of these inhibitors alone in ONS-76 cells (Fig. 2C), suggesting that these inhibitors alone are more efficacious in inducing apoptosis of MYC-amplified MB cells. Together, these results suggest that combination of JQ1 or OTX015 with panobinostat suppresses the cell growth of MYC-amplified MB cells by inducing G2 cell cycle arrest and apoptosis.

### JQ1 and panobinostat synergistically modulate global gene expression in MYC-driven MB cells

**Fig. 3:**
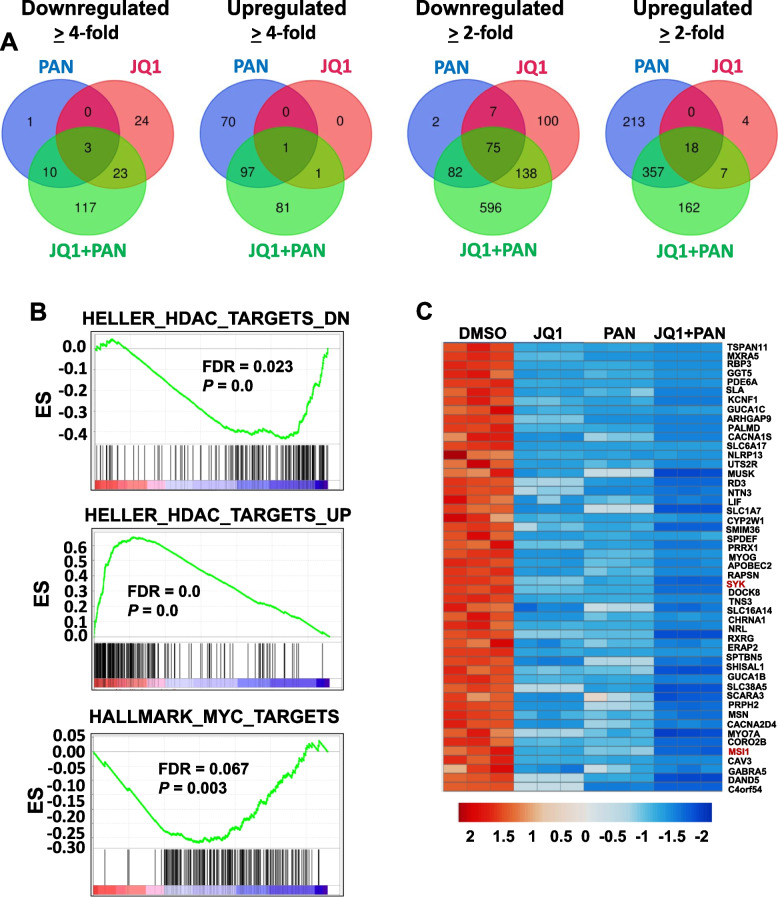
Combined effects of JQ1/PAN on global gene expression. Using RNA-seq, gene expression studies were performed in HD-MB03 cells 24 h after treatment with DMSO (vehicle control), JQ1 (0.5 µM), PAN (10 nM) or the combination of JQ1 and PAN. **A** Venn-diagrams showing number of genes upregulated or downregulated by JQ1 and PAN alone or combined, compared to DMSO. **B** GSE analysis for the modulation of MYC/HDAC target gene sets by the combination of JQ1 and PAN, compared to DMSO. FDR, false discovery rate; ES, enrichment score. **C** Heatmap showing the top 50 genes most significantly downregulated by JQ1-PAN combination treatment

As both BET proteins and HDACs have been shown to modulate global gene transcription [38, 39], we next investigated the combination potential of JQ1 and panobinostat on the overall transcriptional modulation of MYC-amplified HD-MB03 cells. We performed RNA-sequencing in HD-MB03 cells treated with JQ1 and panobinostat alone or combined for 24 h. Using log2 fold-change ≥ 2 as the cutoff, our gene expression data showed that JQ1 upregulated the expression of 3% (18/588) of the genes activated by panobinostat, and panobinostat upregulated the expression of 62% (18/29) genes activated by JQ1 (Fig. 3A). Conversely, JQ1 downregulated the expression of 49.3% (82/166) genes suppressed by panobinostat, and panobinostat downregulated the expression of 25.6% (82/320) genes suppressed by JQ1. As expected, nearly all the genes commonly upregulated or downregulated by JQ1 and panobinostat as single-agent treatments were similarly modulated by the combination of JQ1 and panobinostat (Fig. 3A). The data show that JQ1 and panobinostat alone or combined, activate a common set of genes, and repress a much larger common set of genes, in MB cells.

Notably, our data show that the combination of JQ1 and panobinostat synergistically modulated overall gene expression. For example, using the log2 fold-change cutoff of ≥ 2, gene expression analysis showed that JQ1 alone and panobinostat alone regulated the expression of 320 and 754 target genes, respectively, and the combination of JQ1 and panobinostat modulated the expression of 1435 genes (Fig. 3A). These data were also consistent with the gene expression profile using log2 fold-change cutoff of ≥ 4, where the combination of JQ1 and panobinostat synergistically modulated the expression of more than 300 genes, compared to the expression of 52 and 182 genes respectively modulated by JQ1 and panobinostat alone (Fig. 3A).

In addition, we subjected our expression data to GSE analysis. GSE analysis revealed significant enrichment of MYC and HDAC target gene sets by JQ1-panobinostat combination treatment (Fig. 3B). Further GSE analysis of the other cancer targets identified the gene-enrichment of cell cycle (E2F- and G2M-targets), apoptosis, DNA repair, hypoxia, epithelial-mesenchymal-transition, and stem cell targets by JQ1-panobinostat combination treatment (Supplementary Table S1**)**. Among these, the enrichment of cell cycle and apoptosis associated gene sets supports our prior results (Fig. 2) showing the interaction between JQ1 and panobinostat in inducing G2-cell cycle arrest and apoptosis in MB cells.

### Combination of JQ1 with panobinostat downregulates the expression of SYK and MSI1

**Fig. 4:**
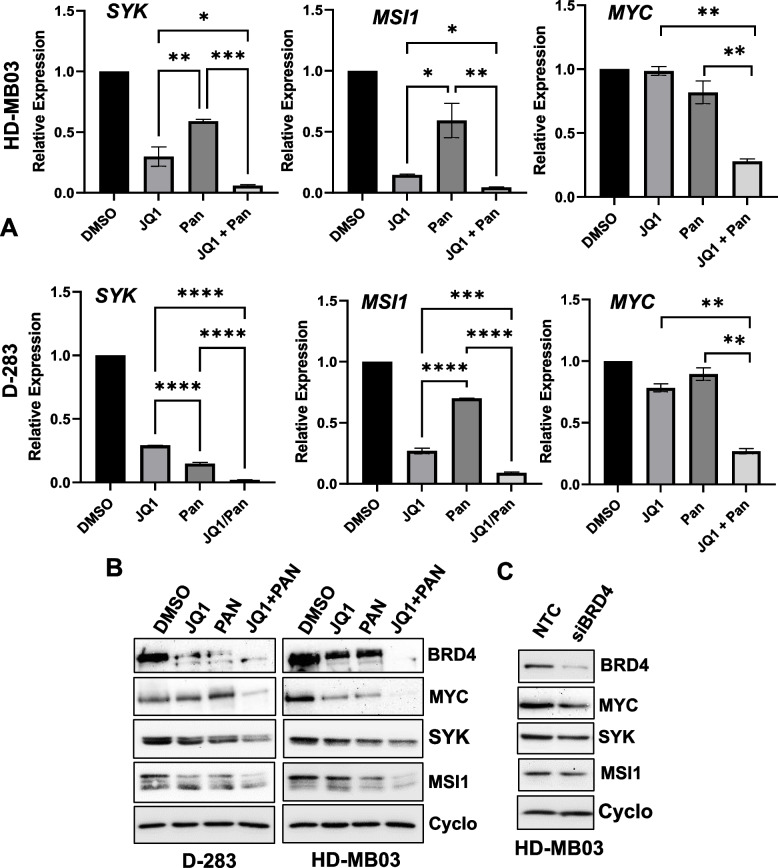
Combined effects of BET and HDAC inhibitors on SYK and MSI1 expression. **A** Quantitative-PCR and **B** western blot analyses for the mRNA and protein expression of BRD4, SYK, MSI1 and MYC in MB HD-MB03 and D-283 cell lines treated for 24 h with 0.5 µM JQ1, 10 nM PAN alone, or both combined. GAPDH and Cyclophillin-B (cyclo)/ Vinculin were used as the loading controls in q-PCR and western blot analyses, respectively. Results are the representative of three independent replicates. Plotted values and error bars represent mean ± SEM. *p < 0.05; **p < 0.01; ***p < 0.001; ****p < 0.0001 (Student-*t*-test). **C** Western blot analysis for the expression of BRD4, SYK, MSI1 and MYC proteins in HD-MB03 cells transfected with scrambled (control-siRNAs) and BRD4-siRNAs (50 nM) for 48 h

We further looked for the top 50 genes most significantly downregulated by the combination. Among these, we identified *SYK* (*spleen tyrosine kinase*) and *MSI1* (*Musashi1*) as the most cancer-relevant genes (Fig. 3C, Supplementary Table S2). Using q-PCR and western blot analyses in HD-MB03 and D-283 MB cell lines, we further confirmed that co-treatment of JQ1 and panobinostat significantly inhibited the expression of SYK and MSI1 at both mRNA and protein levels, compared to individual treatment (Fig. 4A and B). JQ1 or panobinostat alone significantly downregulated the expression of these genes or proteins. In contrast, MYC mRNA was not affected by JQ1 or panobinostat alone, but was significantly downregulated by the JQ1-panobinostat combination (Fig. 4A). Importantly, the expression of BRD4 (a key BET protein outside of the top 50 downregulated genes but especially relevant due to its role as an upstream epigenetic regulator of MYC) and MYC proteins were each downregulated by JQ1 or panobinostat alone, and more profoundly by the combination of these two inhibitors (Fig. 4B).

As the BET inhibitor JQ1 alone significantly downregulates SYK/MSI1 expression (Fig. 4A and B), we further investigated whether the genetic inhibition of BET protein BRD4 also modulates SYK/MSI1 expression. Gene-silencing of BRD4 in HD-MB03 cells by transfection with a pool of BRD4-siRNAs reduced the expression of SYK, MSI1, and MYC proteins, further supporting the epigenetic regulation of SYK and MSI1 by BRD4 in MYC-driven MB cells (Fig. 4C). Together, the data suggest that JQ1 and panobinostat synergistically modulate target gene expression, including MYC/HDAC gene sets and the expression of *SYK* and *MSI1* oncogenes.

**Fig. 5:**
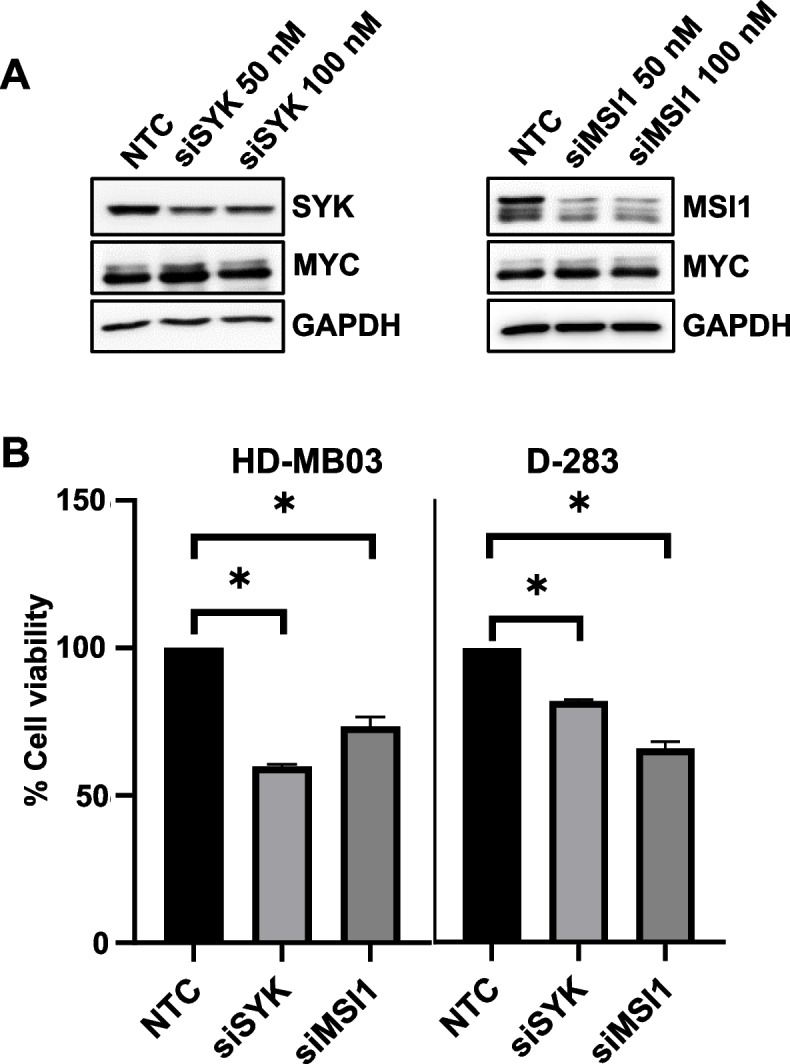
Gene-silencing effects of SYK and MSI1 on MB cell growth and MYC expression. HD-MB03 and D-283 cell lines were transfected with control (NTC), SYK and MSI1 siRNAs (each at 50 nM and 100 nM) for 72 h and subjected to (**A**) western blot (for the expression of SYK or MSI1 and MYC proteins) and (**B**) MTT (cell growth) analyses. GAPDH was used as the loading control in western blot analysis. Results are the representative of three independent experiments. Plotted values and error bars represent mean ± SEM. *p < 0.05 (Student-*t*-test)

Next, we tested if *SYK* and *MSI1* genes, downstream of the BET/HDAC targets, themselves have functional impact on MB cell growth and MYC expression. We therefore determined the effect of siRNA-mediated knockdown of SYK and MSI1 on MYC protein expression and cell growth in HD-MB03 and D-283 MB cell lines. Immunoblot analysis showed that knockdown of both SYK and MSI1 had no effect on MYC expression (Fig. 5A), but significantly reduced cell growth in both cell lines (Fig. 5B).

### Combination of OTX015 with panobinostat reduces tumor growth in MYC-driven MB xenografts

**Fig. 6:**
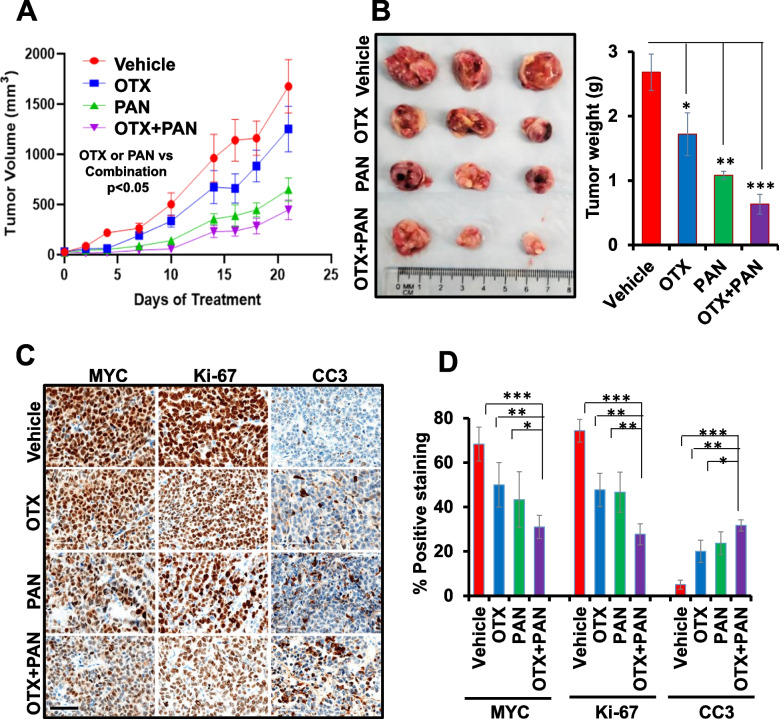
Combined *in vivo* effects of OTX and PAN in subcutaneous MYC-amplified MB-bearing xenografts. **A** Tumor volume measurement of xenografted mice following treatments. The differences between treatment groups represent ANOVA-based comparison of the tumor volumes on 21 days post-treatment. **B** Weight measurement of the tumors (shown in a photo image and plotted in a bar graph) from the last three xenografted mice sacrificed following treatments. Plotted values and error bars represent mean ± SEM. **C** Representative IHC images (40 × magnification with 60 µm scale bar) of MYC, Ki-67 and cleaved-caspase-3 (CC3) in treated xenografts. **D** The percentage of MYC, Ki-67 and CC3 positive cells derived from histology scores was semi-quantified in the tumors (shown in Fig. 6B) of three xenografted mice following 21 days post-treatment of inhibitors. *p < 0.05; **p < 0.01; ***p < 0.001 (ANOVA)

To validate our in vitro results, we further tested the single agent and combined efficacies of BET and HDAC inhibitors in NSG subcutaneous xenograft mice. In this in vivo study, since JQ1 is not being considered in clinical trials because of its short half-life, we utilized the clinically relevant BET inhibitor OTX015, which is in multiple clinical trials in patients with solid tumors or hematologic malignancies [25]. To examine the combined therapeutic potential of OTX015 and panobinostat against MYC-driven MB in vivo, NSG mice were xenografted subcutaneously with HD-MB03 cells and treated with vehicle control, OTX015, panobinostat, and the combination of OTX015 with panobinostat. Our data shows that nearly all mice receiving vehicle control developed tumors of 2 cm^3^ by 21 days post-treatment. Therefore, we considered this time point as the end point for this study. As shown in Fig. 6A and B, 21 days post-treatment with OTX015 or panobinostat alone tumor growth/weight was significantly suppressed, with reductions of 33.5% (by OTX015) and 61.3% (by panobinostat), compared to vehicle control. Combination of OTX015 with panobinostat further significantly suppressed tumor growth/weight by 48% (compared to OTX015) and 17.6% (compared to panobinostat), suggesting the antitumor potential of OTX015 combined with panobinostat, against MYC-driven MB *in vivo*. In addition, treatment with these inhibitors alone or combined did not cause significant changes in the total body weights and histopathology of vital organs between control and treatment groups (Supplementary Fig. S1), suggesting the tolerability of these therapies in mice.

We further examined the effect of OTX015 and panobinostat, alone or combined, on the expression of MYC, proliferation marker Ki-67, and apoptosis marker cleaved-caspase-3 (CC3) in xenografted tumors. Immunohistochemical analyses showed that while OTX015 or panobinostat alone reduced the expression of MYC and Ki-67 and induced the expression of CC3, the combination of OTX015 and panobinostat even more significantly reduced the expression of MYC and Ki-67 and induced the expression of CC3 in xenografted tumors (Fig. 6C and D). The data together suggest that the combination of BET-HDAC inhibitors synergistically inhibits MYC protein expression and proliferation associated markers, thereby blocking the tumor progression of MYC-driven MB *in vivo*.

## Discussion

While the *MYC* oncogene is established as the oncogenic driver in Group 3 MB, it has remained undruggable [38]. Thus, targeting regulatory components of MYC and the downstream signaling pathways that MYC regulates may have great potential therapeutic value. Studies have revealed that damaging germline mutations in known cancer predisposition genes are rare in Group 3 or Group 4 MBs [6], suggesting that dysregulated epigenetic pathways might be critical in the pathogenesis of such MBs. Indeed, recent extensive molecular profiling of primary MBs using transcriptome and methylome profiling identified somatic copy number aberrations and mutations in histone acetyl/methyl transferases, demethylases, and deacetylases [9, 10]. Of special interest, the epigenetic regulators, BET proteins and HDACs, have been shown to regulate the transcription of *MYC* oncogene in MYC-driven MB [16, 17], suggesting that targeting MYC transcription by dual-inhibition of BET proteins and HDACs is a viable strategy. In this study, we show that combined inhibition of BET proteins by JQ1 or OTX015 and HDACs by panobinostat triggered broad antitumor activities in MYC-driven MB, both *in vitro* and *in vivo*. This study provides the first preclinical evidence for the therapeutic potential of dual-targeting BET and HDACs against highly aggressive MB.

With the goal of targeting epigenetic-driven cancers, several epigenetic drugs are being explored as possible therapeutics [39]. Of these, some of the most promising epigenetic anticancer agents are BET inhibitors and HDAC inhibitors. Several BET inhibitors, such as GSK525762 and OTX015, are in multiple clinical trials in patients with solid tumors or hematologic malignancies [25]. Moreover, the recent FDA approval of the HDAC inhibitor panobinostat to treat advanced multiple myeloma [20] highlights the feasibility of developing a BET–HDAC inhibitor combination strategy in the clinic. In this study, we have shown that a combination of JQ1 or OTX015 with panobinostat synergistically induces cell growth inhibition and apoptosis in MB. Importantly, in a subcutaneous mouse model of MYC-driven MB, we have confirmed that OTX015 or panobinostat alone suppresses MB tumor growth, and the combination of these synergistically blocks MB progression. Our study therefore suggests the feasibility of using these inhibitors in combination in MB patients with poor prognoses. This therapeutic strategy has broad relevance for cancer therapy beyond MB, as the findings are consistent with recent reports where the BET inhibitors (JQ1, OTX015 or RVX2135) and the HDAC inhibitors (panobinostat or vorinostat) synergistically inhibit MYC expression and exhibit antitumor efficacies in preclinical models of neuroblastoma, glioblastoma and lymphoma [26–28].

A recent study in MYCN-amplified neuroblastoma showed that JQ1 and panobinostat commonly activate, and more often downregulate, target gene expression in MYCN-amplified neuroblastoma cells [26]. In our gene expression study, we made very similar observations that BET inhibitor JQ1 and HDAC inhibitor panobinostat commonly upregulate, and more often downregulate, target gene expression in MYC-amplified MB cells. Importantly, compared to cells treated with single inhibitors, genes are more substantially modulated (up or downregulated) in cells treated with JQ1 and panobinostat combination. The combination also modulates the expression of a large number of genes not regulated by JQ1 or panobinostat alone. In addition, GSEA analysis reveals that MYC and HDAC target gene sets are among the most significantly enriched by the combination therapy. Together, these results suggest that dual inhibition of BET and HDACs synergistically modulates global gene expression. This synergy may result from the hyperacetylation of histones by panobinostat that increases the affinity of BET proteins binding to open chromatin, thereby enhancing the modulation of gene transcription by BET inhibitors.

Among the top 50 genes (most significantly and synergistically downregulated by JQ1 and panobinostat combination), we identified *SYK* and *MSI1* genes as the most cancer relevant genes, particularly considering their involvement in neurodevelopment [40–43]. Therefore, in this study, *SYK* and *MSI1* oncogenes were selected to further explore their roles in MB tumorigenesis. Interestingly, combination of JQ1 with panobinostat significantly decreased the expression of SYK and MSI1 at both mRNA and protein levels. In addition, combination of JQ1 and panobinostat significantly synergized in reducing MYC protein expression. Importantly, data data from the SYK and MSI1 knockdown experiments showed decreased MB cell growth without affecting MYC expression, indicating that SYK and MSI1 themselves are able to regulate MB tumorigenesis. We therefore hypothesize that reduced expression of SYK and MSI1 is mechanistically important, along with suppressed MYC expression, in the synergistic anticancer effects of these drugs in MB.

To our knowledge, this study is first to demonstrate BET and HDAC are upstream regulators of SYK and MSI1 expression. Beyond this, no evidence exists if SYK and MSI1 have any roles in the context of BET and HDAC driven epigenetic tumor regulation. Nevertheless, there are few evidence suggest that both SYK as a non-receptor tyrosine kinase and MSI1 as an RNA-binding protein can regulate MYC translation/stability by activating/phosphorylating PI3K/AKT signaling and regulating mRNA stability post-transcriptionally, respectively [42, 44, 45]. In this regard, our data with the knockdown of SYK and MSI1 in MB cells showed no effects on MYC protein expression, suggesting that SYK/MSI1 might not have roles in regulating MYC translation/stability in MB. In general, the role of SYK in MB tumorigenesis is largely unexplored. However, SYK kinase plays key roles in promoting tumorigenesis in various other cancers including brain tumor glioblastoma [40, 41]. SYK is well-known oncogene and tumor promoter in leukemia [46]. Activation or overexpression of SYK triggers several signal transduction pathways modulating proliferation, differentiation, and cell survival [40, 46]. More importantly, in addition to hematopoietic cells, SYK is also expressed in brain neuronal cells and regulates cell proliferation and vascular development [47], and therefore could play a key role in MB progression. In comparison, MSI1 has been characterized as a neural stem cell marker, contributes to the maintenance of self-renewal and differentiation of cells, and has been implicated in the tumorigenesis of multiple tumor types including MB [42, 48, 49]. The importance of MSI in maintenance of neural stem cells supports a possible role in MB tumorigenesis. MSI1 has been shown to be expressed in all four MB subgroups, with particularly high expression in Group 3 and 4 tumors [49]. In addition, analysis of MSI1 across a large cohort of MB patients demonstrated that high MSI1 expression is a strong indicator of poor prognosis [49]. Together, the studies based on published reports and our data, suggest that both SYK and MSI1 might play key tumorigenic roles in MYC-driven MB. Further investigation is required to establish mechanism(s) for their tumorigenic roles in the MB tumor microenvironment and to determine if these can be exploited therapeutically.

Although we did not test the BET-HDAC combination strategy in MB orthotopic models, it is evident from preclinical animal studies that BET-HDAC inhibitors including OTX015 and panobinostat, as single agents, can cross the blood–brain-barrier and target brain tumors such as MB and glioblastoma [16, 27, 33, 34]. In addition, in one of our previous MB studies, we have shown that BET inhibitor JQ1 can penetrate brain and block tumor growth in an orthotopic MB mouse model [31]. These findings highlight the feasibility of clinical investigation of this combination strategy in MB. Both subcutaneous and orthotopic/intracranial xenograft models are being used in MB studies. Subcutaneous models are appropriate for initial drug testing and screening as this model allows for easy tumor visualization and quantification, making decisions of treatment initiation less difficult. Orthotopic models of MB require additional features such as modification/optimization of tumor cells in order to image and quantitate tumor growth, or optimization of imaging methods. Experimental parameters to test our findings using patient-derived xenografts (PDX) in a MB orthotopic model are currently under development in our lab.

## Conclusions

In summary, our study demonstrated that targeting epigenetic pathways using BET and HDAC inhibitors has synergistic antitumor potential in MYC-driven MB *in vitro* and *in vivo*. Mechanistically, the combination of BET and HDAC inhibitors synergistically modulates global gene expression, including the expression of MYC and HDAC target gene sets and known oncogenes SYK and MSI1 in MYC-amplified MB cells. Our findings therefore identify a novel potential strategy to inhibit MYC expression in aggressive MB. While further studies using appropriate *in vivo* models are required, our findings highlight a basis for considering this targeted approach as a new therapy for MB.

## Supplementary Information

**Additional file 1**: **Supplementary Table S1.** JQ1 and panobinostat synergistically modulate gene expression. GSE analysis was performed using RNA-sequencing based differential gene expression in HD-MB03 cells 24 h after treatment with control (DMSO) solvent, 0.5 µM JQ1, 10 nM panobinostat, or the combination of JQ1 and panobinostat. GSE analysis generated enriched gene sets, confirming modulation of MYC/HDAC, cell cycle, apoptosis, hypoxia, EMT and stem cell target gene sets by JQ1 and panobinostat alone or in combination. NES, normalized enrichment score; FDR, false discovery rate.

**Additional file 2**: **Supplementary Table S2.** The top 50 genes most significantly downregulated by the combined JQ1-PAN treatment in HD-MB03 cells.

**Additional file 3:** **Fig. S1.** Effects of inhibitors on body weight and histology of the MB xenograft mice. (A) The line graph is Showing the mean body weight of mice following treatment with inhibitors alone or combined as indicated. (B) Histopathology (H&E) of the vital organs of MB xenografts following 21 days post treatment with inhibitors. The images were scanned and captured using digital scanner EVOS Image system at 20x magnification.

## Data Availability

The data generated and/or analyzed during this study are available from the corresponding author on reasonable request.

## Abbreviations

BET: Bromodomain extra-terminal
CI: Combination index
DMSO: Dimethyl-sulfoxide
FBS: Fetal bovine serum
GSEA: Gene set enrichment analysis
HDAC: Histone deacetylase
h: Hour
MB: Medullooblastoma
MTT: 3-(4,5-Dimethylthiazol-2-yl)-2,5-diphenyltetrazolium bromide
MSI1: Musashi1
MYC: V-myc avian mylocytomatosis viral oncogene homolog
MYCN: V-myc avian mylocytomatosis viral oncogene neuroblastoma homolog
nM: Nanomolar
OTX: OTX-015
PAN: Panobinostat
qRT-PCR: Quantitative real-time polymerase chain reaction
RNA-seq: RNA sequencing
siRNA: Silencing-RNA
SYK: Spleen tyrosine kinase
µM: Micromolar
