# Supplementary material for: A novel dual epigenetic approach targeting BET proteins and HDACs in Group 3 (MYC-driven) Medulloblastoma
Source: J Exp Clin Cancer Res. 2022 Nov 11;41:321. doi: 10.1186/s13046-022-02530-y (PMC9650837; doi:10.1186/s13046-022-02530-y)
Supplement: Supplementary file 1 — Additional file 1: Supplementary Table S1. JQ1 and panobinostat synergistically modulate gene expression. GSE analysis was performed using RNA-sequencing based differential gene expression in HD-MB03 cells 24 h after treatment with control (DMSO) solvent, 0.5 µM JQ1, 10 nM panobinostat, or the combination of JQ1 and panobinostat. GSE analysis generated enriched gene sets, confirming modulation of MYC/HDAC, cell cycle, apoptosis, hypoxia, EMT and stem cell target gene sets by JQ1 and panobinostat alone or in combination. NES, normalized enrichment score; FDR, false discovery rate. [file 13046_2022_2530_MOESM1_ESM.docx]

| **Gene Set** | **Size** | **NES** | **P-val** | **FDR q-val** | **NES** | **P-val** | **FDR q-val** | **NES** | **P-val** | **FDR q-val** |
| --- | --- | --- | --- | --- | --- | --- | --- | --- | --- | --- |
| HALLMARK_MYC_TARGETS_V1 | 169 | 1.09 | 0.636 | 0.658 | -2.7 | 0 | 0 | -1.52 | 0.003 | 0.067 |
| KIM_MYC_AMPLIFICATION_  TARGETS_UP | 124 | 0.56 | 1 | 1 | 0.59 | 0.974 | 1 | -1.27 | 0.088 | 0.536 |
| KIM_MYC_AMPLIFICATION_  TARGETS_DN | 33 | -0.72 | 0.834 | 1 | 1.88 | 0 | 0.009 | 1.85 | 0.002 | 0.019 |
| HELLER_HDAC_TARGETS_UP | 191 | 2.54 | 0 | 0 | 2.32 | 0 | 0 | 3.4 | 0 | 0 |
| HELLER_HDAC_TARGETS_DN | 155 | -1.16 | 0.224 | 0.915 | -1.99 | 0 | 0.011 | -2.06 | 0 | 0.023 |
| HELLER_HDAC_TARGETS_  SILENCED_BY_METHYLATION_UP | 255 | 3.1 | 0 | 0 | 2.31 | 0 | 0 | 3.63 | 0 | 0 |
| HELLER_HDAC_TARGETS_  SILENCED_BY_METHYLATION_DN | 149 | -1.11 | 0.293 | 0.983 | -2.09 | 0 | 0.005 | -2.2 | 0 | 0.007 |
| HALLMARK_E2F_TARGETS | 165 | -1.07 | 0.371 | 1 | -2.45 | 0 | 0 | -1.77 | 0 | 0.016 |
| HALLMARK_G2M_CHECKPOINT | 149 | -0.77 | 0.877 | 1 | -2.17 | 0 | 0 | -1.56 | 0.002 | 0.065 |
| WP_DNA_REPAIR_PATHWAYS_  FULL_NETWORK | 73 | -0.82 | 0.78 | 1 | -2.27 | 0 | 0.001 | -1.62 | 0.002 | 0.185 |
| HALLMARK_APOPTOSIS | 98 | 0.9 | 0.8 | 0.733 | 1.75 | 0 | 0.009 | 2.03 | 0 | 0.002 |
| HALLMARK_P53_PATHWAY | 106 | 1.6 | 0 | 0.194 | 1.73 | 0.001 | 0.009 | 2.36 | 0 | 0 |
| HALLMARK_HYPOXIA | 117 | -0.72 | 0.876 | 1 | 1.86 | 0 | 0.004 | 2.24 | 0 | 0 |
| HALLMARK_EPITHELIAL_  MESENCHYMAL_TRANSITION | 100 | -1.29 | 0.119 | 0.611 | 1.56 | 0.01 | 0.047 | 1.87 | 0 | 0.006 |
| JAATINEN_HEMATOPOIETIC_  STEM_CELL_UP | 150 | -1.07 | 0.366 | 1 | -1.46 | 0 | 0.163 | -1.98 | 0 | 0.033 |
| WONG_EMBRYONIC_STEM_CELL_  CORE | 261 | -0.77 | 0.918 | 1 | -2.23 | 0 | 0.002 | -1.75 | 0 | 0.106 |

**Supplementary Table S1.** JQ1 and panobinostat synergistically modulate gene expression. GSE analysis was performed using RNA-sequencing based differential gene expression in HD-MB03 cells 24 h after treatment with control (DMSO) solvent, 0.5 µM JQ1, 10 nM panobinostat, or the combination of JQ1 and panobinostat. GSE analysis generated enriched gene sets, confirming modulation of MYC/HDAC, cell cycle, apoptosis, hypoxia, EMT and stem cell target gene sets by JQ1 and panobinostat alone or in combination. NES, normalized enrichment score; FDR, false discovery rate.

**JQ1 PAN JQ1+PAN**
