# Supplementary material for: A novel dual epigenetic approach targeting BET proteins and HDACs in Group 3 (MYC-driven) Medulloblastoma
Source: J Exp Clin Cancer Res. 2022 Nov 11;41:321. doi: 10.1186/s13046-022-02530-y (PMC9650837; doi:10.1186/s13046-022-02530-y)
Supplement: Supplementary file 2 — Additional file 2: Supplementary Table S2. The top 50 genes most significantly downregulated by the combined JQ1-PAN treatment in HD-MB03 cells. [file 13046_2022_2530_MOESM2_ESM.docx]

**Supplementary Table S2**. The top 50 genes most significantly downregulated by the combined JQ1-PAN treatment in HD-MB03 cells.

|  | **JQ1** |  | **PAN** |  | **JQ1+PAN** |  |
| --- | --- | --- | --- | --- | --- | --- |
| **Gene Name** | Fold Change | P.Value | Fold Change | P.Value | Fold Change | P.Value |
| TSPAN11 | -3.831 | 1.87E-06 | -4.37916 | 1.65E-06 | -9.25379 | 1.20E-06 |
| MXRA5 | -2.98855 | 5.90E-08 | -5.95632 | 2.57E-08 | -8.50699 | 2.53E-08 |
| RBP3 | -3.95755 | 7.77E-08 | -3.88939 | 8.90E-08 | -8.3874 | 4.93E-08 |
| GGT5 | -5.562 | 2.36E-05 | -3.59667 | 3.03E-05 | -8.15727 | 1.86E-05 |
| PDE6A | -3.91011 | 5.87E-08 | -4.11094 | 2.98E-08 | -7.78047 | 2.41E-08 |
| SLA | -3.76888 | 9.52E-06 | -2.60317 | 2.93E-05 | -7.526 | 5.82E-06 |
| KCNF1 | -2.48022 | 6.99E-06 | -3.22411 | 2.65E-06 | -7.33473 | 1.26E-06 |
| GUCA1C | -3.88717 | 6.83E-05 | -5.64799 | 4.75E-05 | -7.27034 | 4.53E-05 |
| ARHGAP9 | -2.28045 | 2.63E-08 | -4.74257 | 6.60E-09 | -7.2387 | 5.38E-09 |
| PALMD | -2.94758 | 4.27E-07 | -3.42033 | 3.33E-07 | -7.18403 | 1.96E-07 |
| CACNA1S | -5.02264 | 7.49E-05 | -2.2082 | 0.000227 | -7.06665 | 7.00E-05 |
| SLC6A17 | -6.90462 | 6.84E-08 | -5.37803 | 7.09E-08 | -7.05714 | 5.71E-08 |
| NLRP13 | -4.06374 | 0.000679 | -5.42627 | 0.000604 | -6.99759 | 0.000546 |
| UTS2R | -4.20796 | 0.00016 | -3.04377 | 0.000229 | -6.93867 | 0.000121 |
| MUSK | -2.46243 | 8.99E-05 | -1.15814 | 0.000507 | -6.82364 | 3.24E-05 |
| RD3 | -1.74781 | 1.23E-07 | -3.12531 | 3.74E-08 | -6.80593 | 1.85E-08 |
| NTN3 | -1.86073 | 1.41E-06 | -4.14682 | 3.51E-08 | -6.624 | 3.76E-08 |
| LIF | -2.92147 | 0.000325 | -2.44846 | 0.000117 | -6.42435 | 4.51E-05 |
| SLC1A7 | -2.94632 | 7.15E-07 | -1.13904 | 9.00E-06 | -6.41605 | 3.52E-07 |
| CYP2W1 | -3.44009 | 0.000408 | -4.21978 | 0.000302 | -6.38218 | 0.000255 |
| SMIM36 | -1.89768 | 2.49E-05 | -2.54604 | 5.21E-06 | -6.28478 | 1.01E-06 |
| SPDEF | -2.76353 | 3.81E-05 | -4.8416 | 5.61E-05 | -6.23483 | 4.56E-05 |
| PRRX1 | -2.26732 | 5.91E-05 | -3.15853 | 3.18E-05 | -6.17044 | 1.98E-05 |
| MYOG | -5.7568 | 1.13E-07 | -2.86653 | 1.59E-07 | -6.15254 | 8.48E-08 |
| APOBEC2 | -3.93448 | 3.81E-07 | -2.68128 | 1.55E-06 | -6.14862 | 2.85E-07 |
| RAPSN | -4.258 | 5.62E-08 | -2.66028 | 9.15E-07 | -6.02076 | 3.73E-08 |
| SYK | -1.93574 | 1.10E-07 | -2.58719 | 1.36E-07 | -5.9155 | 2.44E-08 |
| DOCK8 | -2.20655 | 1.68E-07 | -2.73039 | 8.87E-08 | -5.90531 | 4.28E-08 |
| TNS3 | -3.36823 | 3.25E-07 | -3.23512 | 6.59E-07 | -5.87257 | 3.34E-07 |
| SLC16A14 | -4.56243 | 2.32E-05 | -1.4366 | 0.000305 | -5.79978 | 1.69E-05 |
| CHRNA1 | -5.33972 | 1.43E-05 | -2.98211 | 3.26E-05 | -5.79619 | 1.35E-05 |
| NRL | -3.06454 | 1.80E-05 | -3.10339 | 1.56E-05 | -5.76845 | 9.61E-06 |
| RXRG | -1.6797 | 1.79E-08 | -1.91529 | 5.76E-09 | -5.75095 | 1.69E-09 |
| ERAP2 | -4.03562 | 0.000126 | -2.5145 | 0.000206 | -5.64064 | 8.13E-05 |
| SPTBN5 | -3.90664 | 2.12E-08 | -4.00024 | 1.37E-08 | -5.55294 | 6.70E-09 |
| SHISAL1 | -4.1903 | 4.43E-08 | -1.61369 | 2.70E-07 | -5.53821 | 6.32E-08 |
| GUCA1B | -1.90142 | 1.01E-05 | -1.77961 | 9.17E-06 | -5.50581 | 1.43E-06 |
| SLC38A5 | -2.67233 | 3.46E-07 | -3.12063 | 2.21E-07 | -5.44768 | 1.37E-07 |
| SCARA3 | -1.3095 | 1.24E-06 | -2.48372 | 2.42E-07 | -5.42631 | 7.68E-08 |
| PRPH2 | -2.89083 | 1.66E-05 | -1.18692 | 0.000321 | -5.38223 | 1.03E-05 |
| MSN | -3.22999 | 1.33E-07 | -1.71314 | 3.46E-06 | -5.3747 | 1.44E-07 |
| CACNA2D4 | -2.71445 | 9.66E-06 | -2.32667 | 7.17E-06 | -5.33316 | 1.90E-06 |
| MYO7A | -3.82998 | 2.05E-06 | -2.16342 | 2.23E-05 | -5.29926 | 1.94E-06 |
| CORO2B | -1.41615 | 0.000126 | -1.89739 | 0.000102 | -5.24468 | 1.80E-05 |
| MSI1 | -2.26526 | 1.06E-05 | -2.18485 | 9.79E-06 | -5.16544 | 3.41E-06 |
| CAV3 | -2.42217 | 4.73E-05 | -1.81926 | 0.00011 | -5.15837 | 2.41E-05 |
| GABRA5 | -3.00615 | 5.23E-06 | -3.18842 | 5.19E-06 | -5.0817 | 2.98E-06 |
| DAND5 | -4.51606 | 0.00036 | -1.96249 | 0.002869 | -4.97995 | 0.000304 |
| C4orf54 | -1.18916 | 4.81E-08 | -3.42881 | 3.18E-09 | -4.89178 | 2.45E-09 |
