# Supplementary material for: A novel dual epigenetic approach targeting BET proteins and HDACs in Group 3 (MYC-driven) Medulloblastoma
Source: J Exp Clin Cancer Res. 2022 Nov 11;41:321. doi: 10.1186/s13046-022-02530-y (PMC9650837; doi:10.1186/s13046-022-02530-y)
Supplement: Supplementary file 3 — Additional file 3: Fig. S1. Effects of inhibitors on body weight and histology of the MB xenograft mice. (A) The line graph is Showing the mean body weight of mice following treatment with inhibitors alone or combined as indicated. (B) Histopathology (H&E) of the vital organs of MB xenografts following 21 days post treatment with inhibitors. The images were scanned and captured using digital scanner EVOS Image system at 20x magnification. [file 13046_2022_2530_MOESM3_ESM.docx]

**Mouse body weight (g)**

**B**

**A**

**Vehicle OTX PAN OTX+PAN**

**Lung**

**Liver**

**Kidney**

**Brain**

**Fig. S1. Effects of inhibitors on body weight and histology of the MB xenograft mice.** ***(A)*** *The line graph is* *Showing the mean body weight of mice following treatment with inhibitors alone or combined as indicated.* ***(B)*** *Histopathology (H&E) of the vital organs of MB xenografts following 21 days post treatment with inhibitors. The images were scanned and captured using digital scanner EVOS Image system at 20x magnification.*
